# The physical healthcare of patients in secure hospitals: setting standards for medical equipment

**DOI:** 10.1192/bjo.2021.521

**Published:** 2021-06-18

**Authors:** Abigail Hood, Johanna Andersson, Charlotte Jones, Lisa Gardiner

**Affiliations:** 1 Southern Health NHS Foundation Trust; 2 Ravenswood House

## Abstract

**Aims:**

The increased morbidity and mortality relating to the poor physical health of patients with severe mental illness has repeatedly been an area identified as requiring improvement. Despite this, no national minimum standard has been published around the minimum level of physical health equipment that should be available within an inpatient psychiatric setting.

The aim of this project was to improve and standardise availability of physical health equipment across the five clinical areas within a medium secure inpatient forensic setting, thus enabling optimal and timely medical care and physical examination of patients to occur.

**Method:**

This project used a combination of audit and quality improvement practices. An audit standard was created and current practice was established within the 5 clinical areas of a Medium Secure Forensic Unit. Improvements were made in a systematic and measured way and two audit cycles were completed.

**Result:**

At baseline, the attainment of audit standard ranged from 14-76%. Clinical areas were sharing equipment and there was an inconsistency as to where and how equipment was being stored. Changes implemented included redistribution and reorganisation of equipment which increased attainment to between 48% - 86%. Following this further equipment was ordered and the equipment was separated into that which was required on a daily basis to conduct physical observations and more specialist specific examination equipment. Re-audit found attainment across the five clinical areas being between 90-100%.

**Conclusion:**

Monitoring of physical health within psychiatric inpatient settings is a key area of patient care, and is frequently identified as requiring improvement. Without access to equipment to monitor and assess physical health, this becomes challenging and potentially poorly completed. By standardizing available equipment and furthermore through practical steps such as separating the equipment required on a daily basis and that used less frequently the retention of equipment improved. This enables delivery of high quality, timely and thorough monitoring and assessment of physical health to be achievable.

